# Fractional-order quantum particle swarm optimization

**DOI:** 10.1371/journal.pone.0218285

**Published:** 2019-06-20

**Authors:** Lai Xu, Aamir Muhammad, Yifei Pu, Jiliu Zhou, Yi Zhang

**Affiliations:** School of Computer Science, Sichuan University, Chengdu, Sichuan Province, China; Durham University, UNITED KINGDOM

## Abstract

Motivated by the concepts of quantum mechanics and particle swarm optimization (PSO), quantum-behaved particle swarm optimization (QPSO) was developed to achieve better global search ability. This paper proposes a new method to improve the global search ability of QPSO with fractional calculus (FC). Based on one of the most frequently used fractional differential definitions, the Grünwald-Letnikov definition, we introduce its discrete expression into the position updating of QPSO. Extensive experiments on well-known benchmark functions were performed to evaluate the performance of the proposed fractional-order quantum particle swarm optimization (FQPSO). The experimental results demonstrate its superior ability in achieving optimal solutions for several different optimizations.

## Introduction

Particle swarm optimization (PSO) [1], which is inspired by animal social behaviors, such as birds, was first proposed by Kennedy and Eberhart as a population-based optimization technique. In PSO, the potential solutions, which are called particles, go through the solution space by relying on their own experiences and current best particle. PSO has a competitive performance with the classical Genetic Algorithm (GA) [2], evolutionary programming (EP) [3], evolution strategies (ES) [4], genetic programming (GP)[5] and other classic algorithms. It has attracted increasing attention during recent years thanks to its effectiveness in different optimization problems [6][7][8].

Quantum computer [9] was proposed 30 years ago and the formal definition of the quantum computer was given in the late 1980s. Since the quantum computer has shown its potential in several special problems [10], many efforts were dedicated to this field. Several well-known algorithms were proposed, and Shor’s quantum factoring algorithm was the most famous one in these methods [11].

Inspired by a similar idea, the quantum-behaved particle swarm optimization (QPSO) [12] was introduced in 2004 by Sun et al. to improve the convergence of classical PSO. In quantum space, particles search in the complete solution space and the global optimum is guaranteed. In recent decades, fractional calculus has drawn increasing interests and been a strong branch of mathematical analyses. Furthermore, the random variables in the physical process can be regarded as the substitution of real stochastic motion. As a result, the fractional calculus can be introduced to analyze the physical statuses and procedures of objects in Euclidean space. The fractional differential functions have two features. A fractional differential function is a power function for primary functions, and it is an iterative addition or product of specific functions for the other functions. Meanwhile, it has been proved that many fractional-order models are more suitable to describe the natural phenomena. Based on these observations, fractional calculus has been introduced into many fields such as viscoelastic theory [13], diffusion processing [14] and stochastic fractal dynamics [15]. Most of the researches on fractional-order applications focus on the transient state of physical changes. However, the evolutive procedures of systems are rarely included.

In recent years, QPSO has attracted great attention from many researchers. To balance the global and local searching abilities, Xi et al. proposed a novel QPSO called weighted QPSO (WQPSO)[16]. Jiao et al. proposed a dynamic-context cooperative quantum-behaved particle swarm optimization (CQPSO)[17] for medical image segmentation. Although QPSO and its variants have better performance in some aspects, they do not make full use of the state information during the ergodic process and it is inefficient in hunting global optimum. In this paper, a novel quantum particle swarm optimization with the fractional-order position is proposed. Due to the nonlinear, non-causal and non-stationary characteristics of fractional calculus, searching global optimum can be significantly accelerated [18][19].

The rest of this paper is organized as follows: In section 2, some mathematical background about fractional calculus is introduced. Section 3 presents the basic ideas of PSO and QPSO and the proposed method is also given there. Section 4 demonstrated the experimental results of the proposed method. Finally, Section 5 outlines the conclusion.

## Background theory for fractional calculus

Grünwald-Letnikov (GL) [20], Riemann-Liouville (RL) [21], and Caputo [22] definitions are three different definitions for fractional calculus in Euclidean space. Due to its convenient computational form, GL definition for the fractional derivative is commonly used for engineering problems.

The GL derivative with an order of function is defined as:

$$\begin{array}{l} {}_{a}^{GL}D_{x}^{\alpha }f\left(x\right)=\frac{d^{\alpha }}{{\left[d\left(x-a\right)\right]}^{\alpha }}f\left(x\right)\\ =\underset{N\to \infty }{\lim}\left\{\frac{{\left(\frac{x-a}{N}\right)}^{-\alpha }}{\Gamma \left(-\alpha \right)}\sum_{k=0}^{N-1}\frac{\Gamma \left(k-a\right)}{\Gamma \left(k+1\right)}f\left(x-k\left(\frac{x-a}{N}\right)\right)\right\} \end{array},$$ (1)

where *f*(*x*) is a differintegrable function, [*a*,*x*] is the function duration, and Γ is the gamma function. Here, ${}_{a}^{GL}D_{x}^{\alpha }$ denotes the GL fractional differential operator.

In (1), when *N* is big enough, the limit symbol can be neglected and we can rewrite (1) as:

$$\frac{d^{\alpha }}{dx^{\alpha }}f\left(x\right)≅\frac{x^{-\alpha }N^{\alpha }}{\Gamma \left(-\alpha \right)}\sum_{k=0}^{N-1}\frac{\Gamma \left(k-\alpha \right)}{\Gamma \left(k+1\right)}f\left(x-\frac{kx}{N}\right),$$ (2)

which is a proximate form substituting fractional derivative with multiplication and addition operations [12]. For 1D signal, it has the following expression:

$$\begin{array}{l} \frac{d^{\alpha }}{dx^{\alpha }}f\left(x\right)≅f\left(x\right)+\left(-\alpha \right)f\left(x-1\right)\\ \;+\frac{\left(-\alpha \right)\left(-\alpha +1\right)}{2}f\left(x-2\right)+\ldots \\ \;+\frac{\left(-\alpha \right)\left(-\alpha +1\right)\left(-\alpha +2\right)\ldots \left(-\alpha +n\right)}{n!}f\left(x-n\right) \end{array}.$$ (3)

## Particle swarm optimization with fractional -order position

### Quantum particle swarm optimization

Trajectory analyses in [23] demonstrated that each particle should converge to the corresponding attractor *C*_*i*_, which is given as follows:

$$C_{id}\left(t\right)=a\cdot pb_{id}\left(t\right)+\left(1-a\right)\cdot gb_{d}\left(t\right),a∼U\left(0,1\right),$$ (4)

where *a* = *c*_1_*r*_1_/(*c*_1_*r*_1_+*c*_2_*r*_2_). It can be seen that the local attractor is a stochastic attractor of particle *i* that lies in a hyper-rectangle with *pb*_*id*_ and *gb*_*d*_ being two ends of its diagonal.

Based on the convergence analysis of PSO [24], inspired by the theory of quantum physics, Sun et al. studied the convergence behavior of PSO and proposed a novel PSO model from quantum mechanics abbreviated as QPSO [25]. Based on the Delta potential, the quantum behavior of particles are considered. In the framework of quantum time-space, the quantum state of a particle can be defined by a wave function *ψ*(*x*,*t*). In 3-D space, *ψ*(*x*,*t*) is given as

$${\left|\psi \right|}^{2}dxdydz=Qdxdydz,$$ (5)

where Q is the probability that measures the particle’s location in the 3-D space. As a probability density function, we have

$$\int_{-\infty }^{+\infty }{\left|\psi \right|}^{2}dxdydz=\int_{-\infty }^{+\infty }Qdxdydz=1.$$ (6)

The normalized version of *ψ* can be given as:

$$\psi \left(y\right)=\frac{1}{\sqrt{L}}e^{-\left|y\right|/L},$$ (7)

As a result, Q and the corresponding distribution function F can be obtained as:

$$Q\left(y\right)={\left|\psi \left(y\right)\right|}^{2}=\frac{1}{L}e^{-2\left|y\right|/L},$$ (8)

And

$$F\left(X_{id}\left(t+1\right)\right)=1-e^{-\frac{2\left|p_{id}\left(t\right)-X_{id}\left(t+1\right)\right|}{L_{id}\left(t\right)}},$$ (9)

where *L*_*id*_(*t*) denotes the standard deviation, which describes the search range of each particle. The position of the particle can be obtained by Monte Carlo method with the following formula:

$$s=\frac{1}{L}u=\frac{1}{L}e^{-2\left|y\right|/L},u=rand\left(0,1\right),$$ (10)

where *s* denotes a random constant, which is uniformly distributed on U(0,1L).

Then, *u* = *e*^−2|*y*|/*L*^. Let *y* = *x*−*c*, we have

$$x=c\pm \frac{L}{2}\ln\left(\frac{1}{u}\right).$$ (11)

The convergence condition of PSO is given by:

x→c,whent→∞. (12)

Let *L* be the function of time, we have:

L=L(t)

L→0,whent→0. (13)

With (13), we have the iterative version of *i*-th multidimensional particle as follows

$$X_{id}\left(t+1\right)=C_{id}\left(t\right)\pm \frac{L_{id}\left(t\right)}{2}\ln\left(\frac{1}{u}\right).$$ (14)

A global point called mean best position is introduced to evaluate *L*_*id*_(*t*). The global point, which is denoted by *mbest*, can be computed as the mean of the *pbest* positions of all particles, which can be given as:

$$\begin{array}{l} mbest\left(t\right)=\left(mbest_{1}\left(t\right),mbest_{2}\left(t\right),\ldots ,mbest_{d}\left(t\right)\right)\\ \;=\frac{1}{n}\sum_{i=1}^{n}p_{i1}\left(t\right),\frac{1}{n}\sum_{i=1}^{n}p_{i2}\left(t\right),\ldots ,\frac{1}{n}\sum_{i=1}^{n}p_{id}\left(t\right) \end{array}.$$ (15)

The values of *L*_*id*_(*t*) is calculated by:

$$L_{id}\left(t\right)=2\cdot \beta \cdot \left|m_{d}\left(t\right)-X_{id}\left(t\right)\right|.$$ (16)

Finally, the position can be given by:

$$X_{id}\left(t+1\right)=C_{id}\left(t\right)\pm \beta \cdot \left|mbest_{d}-X_{id}\left(t\right)\right|\ln\left(\frac{1}{u}\right),$$ (17)

where parameter *β* is step size, which is utilized to control the convergence speed. *rand* is a random number with a range of 0 to 1, which is the deciding factor of “±” in (17).

Table 1 illustrates the main steps of QPSO.

**Table 1:** The main steps of QPSO.

Alogtihm2
Initialize QPSO parameters; **Repeat** **For all** particles **do** compute *f* If *f*(*x*_*i*_)<*f*(*pb*_*i*_) *pb*_*i*_ = *X*_*i*_ End If *f*(*pb*_*i*_)<*f*(*gb*) *gb* = *pb*_*i*_ End Calculate Q using (19) If *rand*>0.5 $X_{id}\left(t+1\right)=C_{id}\left(t\right)+\beta \cdot \left|mbest_{d}-X_{id}\left(t\right)\right|\cdot \ln\left(\frac{1}{u}\right)$ Else $X_{id}\left(t+1\right)=C_{id}\left(t\right)-\beta \cdot \left|mbest_{d}-X_{id}\left(t\right)\right|\cdot \ln\left(\frac{1}{u}\right)$ **End** *t* = *t*+1 **Unti**l stopping criteria

### QPSO with the fractional-order position

It is well known that fractional calculus has a remarkable long-term memory characteristic [26]. From the definition of Grünwald-Letnikov in (1), it can be seen that fractional derivative is computed with all historical states and it is naturally suitable for the iterative procedure of intelligent optimization algorithms. For examples, Pires E.J.S introduced fractional calculus theory into the updated formula of particle swarm optimization algorithm [27].

To further improve the speed and accuracy of convergence of QPSO, in this section, the proposed QPSO with the fractional-order position is detailed. Initially, the original position is rearranged to modify the order of the position derivative, which can be derived as:

$$X_{id}\left(t+1\right)=C_{id}\left(t\right)+\beta \cdot \ln\left(\frac{1}{u}\right)\cdot \left(mbest_{d}-X_{id}\left(t\right)\right)\;\left(rand>0.5,mbest_{d}>X_{id}\left(t\right)\right),$$ (18)

$$X_{id}\left(t+1\right)=C_{id}\left(t\right)+\beta \cdot \ln\left(\frac{1}{u}\right)\cdot \left(X_{id}\left(t\right)-mbest_{d}\right)\;\left(rand>0.5,mbest_{d}<X_{id}\left(t\right)\right),$$ (19)

$$X_{id}\left(t+1\right)=C_{id}\left(t\right)-\beta \cdot \ln\left(\frac{1}{u}\right)\cdot \left(mbest_{d}-X_{id}\left(t\right)\right)\;\left(rand<0.5,mbest_{d}>X_{id}\left(t\right)\right),$$ (20)

$$X_{id}\left(t+1\right)=C_{id}\left(t\right)-\beta \cdot \ln\left(\frac{1}{u}\right)\cdot \left(X_{id}\left(t\right)-mbest_{d}\right)\;\left(rand<0.5,mbest_{d}<X_{id}\left(t\right)\right),$$ (21)

(23) and (26) can be uniformly rewritten as:

$$X_{id}\left(t+1\right)-X_{id}\left(t\right)=C_{id}\left(t\right)+\beta \cdot \ln\left(\frac{1}{u}\right)\cdot mbest_{d}-\left(\beta \cdot \ln\left(\frac{1}{u}\right)\pm 1\right)X_{id}\left(t\right).$$ (22)

The left side of (22) is the discrete version of the derivative with *α* = 1 and we can extend (22) to a generalized version, leading to the following fractional-order expression

$$D^{\alpha }\left(X_{id}\left(t+1\right)\right)=C_{id}\left(t\right)+\beta \cdot \ln\left(\frac{1}{u}\right)\cdot mbest_{d}-\left(\beta \cdot \ln\left(\frac{1}{u}\right)\pm 1\right)X_{id}\left(t\right),$$ (23)

when *rand*>0.5,*mbest*_*d*_>*X*_*id*_(*t*) and *rand*<0.5,*mbest*_*d*_<*X*_*id*_(*t*).

Similarly, for *rand*>0.5,*mbest*_*d*_<*X*_*id*_(*t*) and *rand*<0.5,*mbest*_*d*_>*X*_*id*_(*t*), we have

$$D^{\alpha }\left(X_{id}\left(t+1\right)\right)=C_{id}\left(t\right)-\beta \cdot \ln\left(\frac{1}{u}\right)\cdot mbest_{d}+\left(\beta \cdot \ln\left(\frac{1}{u}\right)\pm 1\right)X_{id}\left(t\right).$$ (24)

Previous researches have demonstrated that while the order *α* of the derivative is set to [0,1], it will introduce a smoother variation and prolong memory effect, which may lead to a better performance than original integral-order method [12][13]. To study the behavior of the proposed fractional-order strategy, a set of functions are tested and the order *α* is set to range from 0 to 1 with step size of Δ*α* = 0.1. To simplify the computational complexity, we usually truncate (3) and only use the first four terms, so we have

$$\begin{array}{l} D^{\alpha }\left(X_{id}\left(t+1\right)\right)=X_{id}\left(t+1\right)-\alpha X_{id}\left(t\right)\\ \;-\frac{1}{2}\alpha \left(1-\alpha \right)X_{id}\left(t-1\right)\\ \;-\frac{1}{6}\alpha \left(1-\alpha \right)\left(2-\alpha \right)X_{id}\left(t-2\right)\\ \;-\frac{1}{24}\alpha \left(1-\alpha \right)\left(2-\alpha \right)\left(3-\alpha \right)X_{id}\left(t-3\right) \end{array}.$$ (25)

Then, (23) can be modified to

$$\begin{array}{l} X_{id}\left(t+1\right)=C_{id}\left(t\right)+\beta \cdot \ln\left(\frac{1}{u}\right)\cdot mbest_{d}\\ \;-\left(\beta \cdot \ln\left(\frac{1}{u}\right)\pm 1-\alpha \right)X_{id}\left(t\right)+XX_{id}\left(t\right) \end{array},$$ (26)

and (24) can be also rewritten as

$$\begin{array}{l} X_{id}\left(t+1\right)=C_{id}\left(t\right)-\beta \cdot \ln\left(\frac{1}{u}\right)\cdot mbest_{d}\\ \;+\left(\beta \cdot \ln\left(\frac{1}{u}\right)\pm 1+\alpha \right)X_{id}\left(t\right)+XX_{id}\left(t\right) \end{array},$$ (27)

where

$$\begin{array}{l} XX_{id}\left(t\right)=\frac{1}{2}\alpha \left(1-\alpha \right)X_{id}\left(t-1\right)\\ \;+\frac{1}{6}\alpha \left(1-\alpha \right)\left(2-\alpha \right)X_{id}\left(t-2\right)\\ \;+\frac{1}{24}\alpha \left(1-\alpha \right)\left(2-\alpha \right)\left(3-\alpha \right)X_{id}\left(t-3\right) \end{array}.$$ (28)

It can be seen that from (23) and (24), the position updating of particles depends not only on the position of the previous particle but also on the historical position of the particle in different points in time. The position updating of particles is the result of long-term memory, which can protect the population distribution and diversity to a certain extent. The flowchart of the proposed quantum-behaved swarm optimization with the fractional position (FQPSO) is shown in Table 2.

**Table 2:** The main steps of FQPSO.

Alogtihm3
Initialize FQPSO parameters; Initialize population: random *X*_*i*_ For each particle *i*∈[*i*,*s*] compute *f* If *f*(*x*_*i*_)<*f*(*pb*_*i*_) *pb*_*i*_ = *X*_*i*_ End If *f*(*pb*_*i*_)<*f*(*gb*) *gb* = *pb*_*i*_ End Calculate Q using the equation If *rand*>0.5,*mbest*_*d*_<*X*_*id*_(*t*) or *rand*<0.5,*mbest*_*d*_>*X*_*id*_(*t*) $X_{id}\left(t+1\right)=C_{id}\left(t\right)+\beta \cdot \ln\left(\frac{1}{u}\right)\cdot mbest_{d}-\left(\beta \cdot \ln\left(\frac{1}{u}\right)\pm 1-\alpha \right)X_{id}\left(t\right)+XX_{id}\left(t\right)$ Else If *rand*>0.5,*mbest*_*d*_>*X*_*id*_(*t*) or *rand*<0.5,*mbest*_*d*_<*X*_*id*_(*t*) $X_{id}\left(t+1\right)=C_{id}\left(t\right)-\beta \cdot \ln\left(\frac{1}{u}\right)\cdot mbest_{d}+\left(\beta \cdot \ln\left(\frac{1}{u}\right)\pm 1+\alpha \right)X_{id}\left(t\right)+XX_{id}\left(t\right)$ **End** *t* = *t*+1 **Unti**l termination criterion is satisfied

## Experiments

### Experimental setup

To validate the performance of the proposed FQPSO, 8 benchmark functions [28–30] listed in Table 3 were used to compare FQPSO with PSO and QPSO under the same maximum function evaluations (FEs). For FQPSO, the order was set to from 0.1 to 0.9 with step 0.1. Firstly, to investigate the impact of a fractional position in the proposed algorithm, we use FQPSO with different fractional-orders to compare to QPSO. Then, the best results of FQPSO were used for comparison with other variants of PSO including PSO [31], QPSO, PSO with both chaotic sequences and crossover operation(CCPSO) [32], naive PSO(NPSO) [33] and moderate-random-search strategy PSO(MRPSO) [34]. The parameters of the compared algorithms were set as recommended in the original references. Since the impact of population size on the performance of PSO-based methods is of the minimum significance [35], all experiments in this research were performed with a population size of 20. [34].

**Table 3:** Benchmark test functions.

F	Formula	Range	*X*_max_	*f*_min_	X*
*f*_1_	$\sum_{i=1}^{n}x_{i}^{2}$	[-100,100]	100	0	0
*f*_2_	$\sum_{i=1}^{n}{\left(\sum_{j=1}^{i}x_{j}\right)}^{2}$	[-100,100]	100	0	0
*f*_3_	$\sum_{i=1}^{n}i\cdot x_{i}^{2}$	[-100,100]	100	0	0
*f*_4_	$\sum_{i=1}^{n}\left|x_{i}\right|+\prod_{i=1}^{n}\left|x_{i}\right|$	[-10,10]	100	0	0
*f*_5_	${\sum_{i=1}^{n}\left(x_{i}\right)}^{2}+{\prod_{i=1}^{n}\left(x_{i}\right)}^{2}$	[-10,10]	100	0	0
*f*_6_	$\sum_{i=1}^{n}\left(x_{i}^{2}-10\;\cos\left(2\pi x_{i}\right)+10\right)$	[-100,100]	100	0	0
*f*_7_	$\begin{array}{l} \sum_{i=1}^{n}\left(\sum_{k=0}^{20}{\left(0.5\right)}^{k}\cos\left(2\pi {\left(3\right)}^{k}\left(x_{i}+0.5\right)\right)\right)\\ -n\sum_{k=0}^{20}\left({\left(0.5\right)}^{k}\cos\left(2\pi \cdot 3^{k}\cdot 0.5\right)\right) \end{array}$	[-5.12,5.12]	5.12	0	0
*f*_8_	−20exp(−0.2(1n∑i=1nxi2)12)−exp(1n∑i=1ncos2πxi)+20+e	[-5.12,5.12]	32	0	0

X* denotes the global optimum.

The parameters of the compared algorithms were set as recommended in the original references. Since the impact of population size on the performance of PSO-based methods is of the minimum significance [35], all experiments in this research were performed with a population size of 20. *β* is computed according to the following formula:

$$\beta \left(t\right)=\left(\beta_{0}-\beta_{1}\right)\left(t_{\max}-t\right)/t_{\max}+\beta_{1},$$ (29)

where *β*_0_ = 0.8, *β*_1_ = 0.6, *t* is the current number of iterations and *t*_max_ is the maximum number of iterations [36].

### Testing FQPSO with different fractional-order

**Fig 1:**
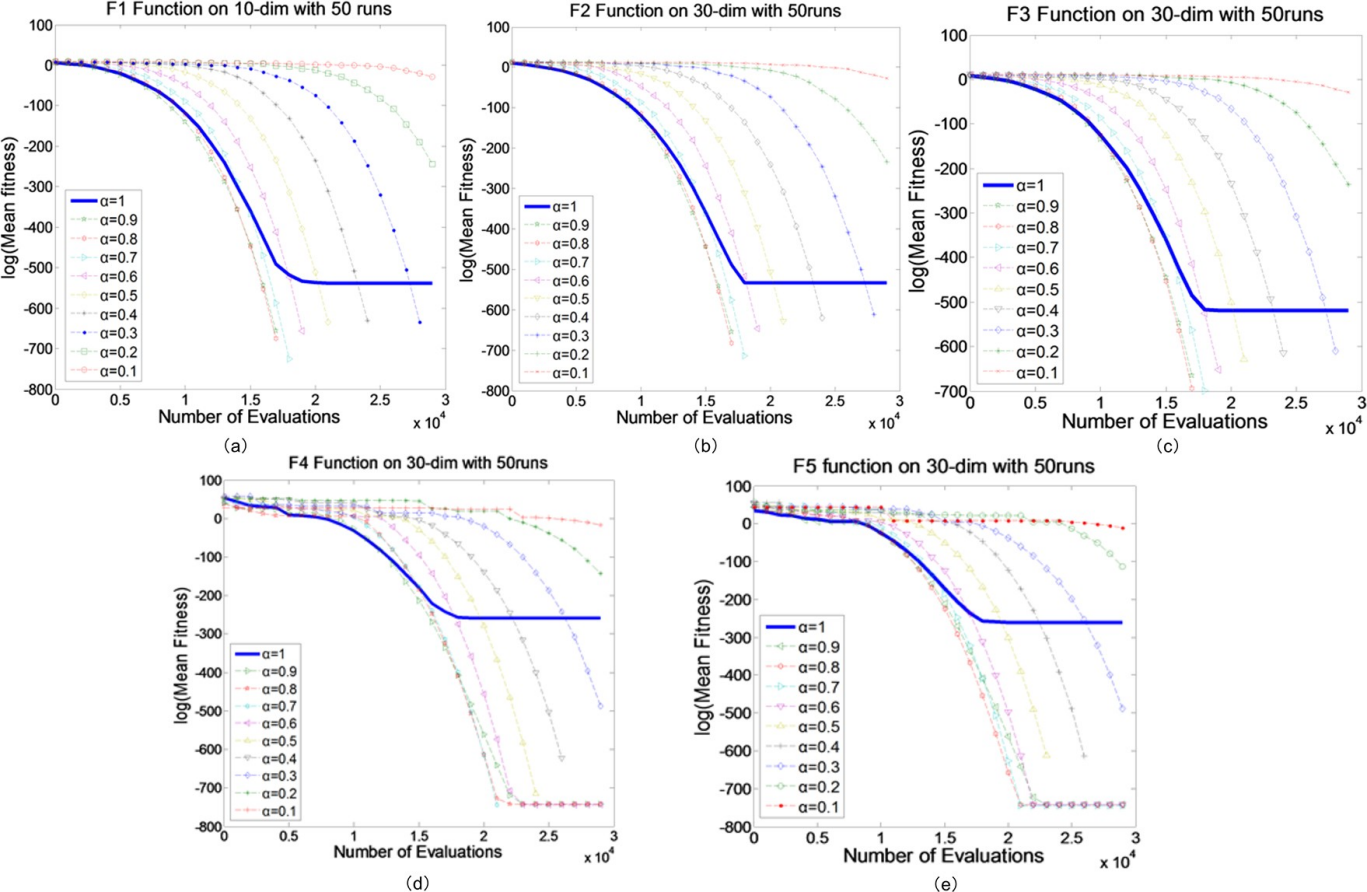
Comparison between FQPSO with different fractional-order on Group 1. (a) *f*_1_, (b) *f*_2_, (c) *f*_3_, (d) *f*_4_, (e) *f*_5_.

**Fig 2:**
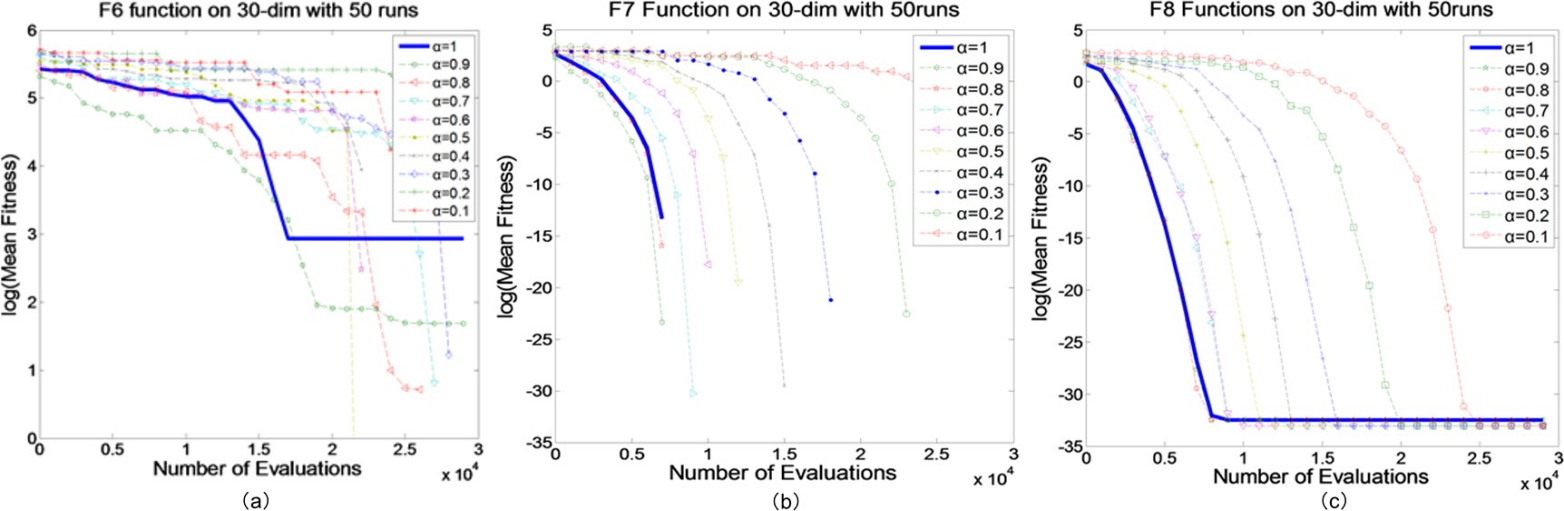
Comparison between FQPSO with different fractional-order on Group 2. (a) *f*_6_, (b) *f*_7_, (c) *f*_8_.

Since QPSO is a stochastic algorithm, it will lead to a different trajectory convergence every time. Therefore, the simulations were performed 50 times with each value in the parameter set *α* = {0,0.1,0.2,…,1}. In Figs 1 and 2, the result is given for the adopted optimization functions *f*_*j*_, *j* = 1,2,…,8. To show the gains achieved by our proposed algorithm, three groups of the experiments were performed. In unimodal functions (*f*_1_-*f*_5_, Group 1) and multimodal functions (*f*_6_-*f*_8_, Group 2) tests, the maximum numbers of FEs were set to 10000, 30000 and 100000, for 10-D, 30-D and 100-D problems, respectively. In the results, we provided the best results and the mean results. The final results over 50 runs of FQPSO are summarized in Tables 4–7.

**Table 4:** Comparison between FQPSOs with different fractional-order on function 1–2.

Fractional-order		*f*_1_			*f*_2_		
		Dim = 10 FEs = 10000	Dim = 30 FEs = 30000	Dim = 100 FEs = 30000	Dim = 10 FEs = 10000	Dim = 30 FEs = 30000	Dim = 100 FEs = 30000
*α* = 1	Best Mean	5.6324e-267 4.5321e-265	2.3453e-241 1.5837e-239	2.9833e-230 3.5636e-231	3.7568e-258 1.8379e-255	1.7033e-237 6.8877e-234	1.4328e-163 4.5673e-158
*α* = 0.9	Best Mean	0 0	0 0	5.3234e-269 4.5639e-267	0 0	0 0	5.3293e-204 6.3214e-201
*α* = 0.8	Best Mean	0 0	0 0	7.3535e-248 6.4356e-246	0 0	0 0	8.5313e-198 4.2314e-197
*α* = 0.7	Best Mean	0 0	0 0	5.3623e-242 7.5323e-239	0 0	0 0	5.3241e-188 3.1235e-185
*α* = 0.6	Best Mean	0 0	0 0	7.4342e-197 6.4329e-194	0 0	0 0	8.4232e-163 5.3123e-161
*α* = 0.5	Best Mean	0 0	0 0	5.3252e-146 5.9753e-143	0 0	0 0	4.3242e-141 8.5223e-140
*α* = 0.4	Best Mean	0 0	0 0	9.5332e-108 5.4256e-99	0 0	0 0	5.3213e-111 6.4132e-108
*α* = 0.3	Best Mean	0 0	0 0	6.5352e-56 5.953e-50	0 0	0 0	8.5231e-66 5.3145e-49
*α* = 0.2	Best Mean	2.1613e-236 6.6966e-250	5.1257e-106 2.2847e-100	6.4235e-18 3.4562e-11	2.1552e-152 7.0445e-145	1.0355e-105 2.2098e-98	1.2345e-16 8.5242e-08
*α* = 0.1	Best Mean	2.613e-53 1.2778e-45	3.5677e-16 3.9080e-12	4.5712e-08 3.4564e-05	1.4244e-36 2.0523e-24	4.5673e-15 2.4097e-11	5.3113e-06 4.5313e-04

**Table 5:** Comparison between FQPSOs with different fractional-order on function 3–4.

Fractional-order		*f*_3_			*f*_4_		
		Dim = 10 FEs = 10000	Dim = 30 FEs = 30000	Dim = 100 FEs = 30000	Dim = 10 FEs = 10000	Dim = 30 FEs = 30000	Dim = 100 FEs = 30000
*α* = 1	Best Mean	6.325e-259 7.424e-255	1.0111e-228 1.2325e-224	4.3529e-194 7.5332e-179	1.4622e-248 1.2011e-215	6.2412e-111 2.6011e-99	6.4353e-56 8.4224e-41
*α* = 0.9	Best Mean	0 0	0 0	8.4243e-223 6.5324e-215	0 0	3.5000e-323 5.9000e-323	7.5224e-75 6.3243e-71
*α* = 0.8	Best Mean	0 0	0 0	9.5352e-245 5.3563e-228	0 0	1.0000e-323 4.9000e-324	8.4242e-77 4.2412e-73
*α* = 0.7	Best Mean	0 0	0 0	7.5324e-237 6.4256e-229	0 0	0 0	6.3242e-65 5.4224e-61
*α* = 0.6	Best Mean	0 0	0 0	8.5363e-185 6.4353e-178	0 0	0 1.000e-323	9.4245e-39 6.2345e-36
*α* =0.5	Best Mean	0 0	0 0	9.5363e-166 7.5352e-161	0 0	0 0	4.2135e-34 1.2356e-33
*α* = 0.4	Best Mean	0 0	0 0	8.4256e-154 9.5324e-151	6.2616e-251 5.5247e-243	0 0	5.2214e-27 5.6241e-21
*α* = 0.3	Best Mean	0 0	0 3.78e-321	6.3245e-134 5.4242e-131	2.6343e-147 9.3570e-141	3.7503e-228 2.8574e-221	7.5213e-18 5.2134e-15
*α* = 0.2	Best Mean	2.1613e-236 6.6966e-220	9.7128e-104 4.7649e-100	5.3523e-67 6.3213e-60	1.5234e-74 2.0325e-68	3.9143e-73 5.1233e-69	5.6231e-12 7.5234e-07
*α* = 0.1	Best Mean	1.2583e-45 5.6267e-39	7.5296e-15 7.9333e-12	3.4525e-06 5.3256e-03	5.9892e-30 2.2309e-25	6.6300e-13 4.2332e-10	4.3241e-08 3.3413e-05

**Table 6:** Comparison between FQPSOs with different fractional-order on function 5–6.

Fractional-order		*f*_5_			*f*_6_		
		Dim = 10 FEs = 10000	Dim = 30 FEs = 30000	Dim = 100 FEs = 30000	Dim = 10 FEs = 10000	Dim = 30 FEs = 30000	Dim = 100 FEs = 30000
*α* = 1	Best Mean	9.397e-189 3.5943e-185	1.1027e-154 1.1027e-155	5.4324e-109 4.5632e-110	0.9950 1.5919	11.9395 16.0250	32.4524 56.3234
*α* = 0.9	Best Mean	0 0	0 0	6.4242e-134 5.6321e-130	1.7764e-15 0.1090	0.9950 6.6484	1.7432 4.5252
*α* = 0.8	Best Mean	0 0	0 0	3.4245e-129 5.6324e-126	0 0	0.4517 2.2933	0.9985 10.4245
*α* = 0.7	Best Mean	0 0	0 0	8.7432e-119 4.5256e-115	0 0	0.0297 2.4696	0.5943 3.4255
*α* = 0.6	Best Mean	0 0	0 0	6.5241e-89 5.3241e-88	0 0	0.5342 12.4468	1.4252 9.3245
*α* = 0.5	Best Mean	0 0	0 0	8.5352e-82 5.4232e-77	2.456e-08 0.01413	4.5314e-06 6.4245e-04	0.04255 0.4256
*α* = 0.4	Best Mean	0 0	0 0	9.4256e-74 6.6322e-72	0.1389 1.5126	6.6789 56.6533	11.3352 57.3241
*α* = 0.3	Best Mean	4.2206e-251 2.3626e-231	5.6014e-317 1.6254e-286	5.3214e-66 5.3242e-65	0.3100 0.3394	0.6789 3.3932	16.4252 54.1343
*α* = 0.2	Best Mean	1.5239e-120 2.9750e-102	2.4207e-99 1.2928e-77	1.2345e-49 2.4562e-46	1.6976e-10 2.1109e-09	10.2080 185.937	26.4952 192.345
*α* = 0.1	Best Mean	4.5632e-42 2.9225e-30	1.2071e-16 1.6344e-06	9.5224e-08 3.4521e-04	1.0415 16.0174	36.4428 66.9430	86.4211 211.245

**Table 7:** Comparison between FQPSOs with different fractional-order on function 7–8.

Fractional-order		*f*_7_			*f*_8_		
		Dim = 10 FEs = 10000	Dim = 30 FEs = 30000	Dim = 100 FEs = 30000	Dim = 10 FEs = 10000	Dim = 30 FEs = 30000	Dim = 100 FEs = 30000
*α* = 1	Best Mean	7.1054e-15 9.9476e-15	2.5251e-10 4.8798e-07	8.5322e-08 5.4252e-07	1.4622e-248 1.2011e-215	7.9936e-15 7.9936e-15	4.5231e-06 3.4251e-05
*α* = 0.9	Best Mean	1.9257e-17 1.8609e-13	5.6302e-11 3.7460e-08	6.5232e-15 7.5323e-11	0 0	4.4409e-15 6.9278e-15	2.3451e-07 4.4123e-06
*α* = 0.8	Best Mean	4.9016e-25 5.6360e-18	5.4000e-10 1.1253e-07	7.4213e-19 9.3421e-15	0 0	1.6409e-15 5.1514e-15	8.4222e-07 5.3245e-06
*α* = 0.7	Best Mean	8.3079e-33 1.1100e-22	5.4000e-20 8.0348e-15	6.4231e-24 5.3313e-19	0 0	4.4409e-15 4.4708e-15	6.4134e-07 5.3121e-06
*α* = 0.6	Best Mean	5.0220e-44 2.4418e-26	3.4005e-12 1.0209e-08	1.2334e-14 5.4134e-13	0 0	4.4409e-15 4.4409e-15	4.3311e-07 4.2134e-06
*α* = 0.5	Best Mean	6.5183e-43 9.7154e-22	1.6486e-11 2.2779e-09	7.4231e-09 9.3134e-06	0 0	4.4409e-15 4.4409e-15	1.2134e-08 5.4131e-06
*α* = 0.4	Best Mean	4.765e-28 8.98017e-13	3.1412e-17 3.1149e-14	8.4255e-10 5.3424e-08	6.2616e-251 5.5247e-243	4.4409e-15 4.4409e-15	3.4131e-07 3.4111e-06
*α* = 0.3	Best Mean	2.0456e-16 1.1142e-14	3.9874e-13 1.0676e-10	8.4325e-12 4.5231e-10	2.6343e-147 9.3570e-141	3.7503e-15 2.8574e-15	5.3314e-08 4.3141e-07
*α* = 0.2	Best Mean	3.1005e-19 6.01e-13	4.2733e-17 2.6818e-12	3.4112e-05 1.2144e-03	1.5234e-74 2.0325e-68	6.7564e-15 6.9345e-15	6.5131e-03 4.5131e-01
*α* = 0.1	Best Mean	0.004777 0.0865	0.0813 0.7666	0.1133 1.2134	5.9892e-30 2.2309e-25	7.9835e-15 8.2343e-15	0.1314 1.2144

Fig 1 shows the performance of FQPSO with different fractional-orders in Group 1. *f*_1_, a Sphere function, is the most widely used unimodal test function. Compared with algorithms with integer-order position, FQPSO shows the best results for this function. Similar results were obtained for other unimodal functions. The improvements achieved by FQPSO on these unimodal functions suggest that fractional-order methods are better at a fine-gained search than integer-order ones. However, it is also worth noting that the performances of FQPSO algorithms with orders 0.1 and 0.2 were not better than integer-order. The reason is that (35) is just an approximation of *D*^*α*^ and the approximation accuracy of *D*^0.1^ and *D*^0.2^ is not good enough. From Fig 1, we can see that most FQPSO methods’ convergence accuracies are better than QPSO. For 10-D and 30-D problems in function 1, 2 and 3 showed in Fig 1A, 1B and 1C and Tables 5 and 6, when 0.3≤*α*≤0.9, the convergence accuracies are better than QPSO. For 10-D and 30-D problems in function 4, the convergence accuracies of are better than QPSO, when 0.4≤*α*≤0.9. For 10-D and 30-D problems in function 5, the convergence accuracies of are better than QPSO, when 0.2≤*α*≤0.9. Tables 5–7 also show that the convergence accuracies are better than QPSO in function 1, 2, 3, 4 and 5 when 0.7≤*α*≤0.9 for100-D problems.

In general, we can always find an appropriate fractional order so that the convergence accuracy of the algorithm is better than the integer order algorithm in Group 1.

In Fig 2, for *f*_6_, *f*_7_ and *f*_8_, the numbers of local minima will increase dramatically as the dimension of the function raises. In this part, we mainly investigated the capability of global searching. *f*_6_ is the generalized Rastrigin’s function, which is the most widely used test multimodal functions in PSO algorithm, and tends to be trapped by local minimums. Considering more orders to search in the solution space, FQPSO gets more favorable results than the compared algorithms. *f*_7_ is the Ackley function and according to the results in Table 8, the performances of FQPSO have little changes with the variation of dimension and achieve the best results on each dimension. Function *f*_8_ is the Weierstrass function, which is continuous everywhere, but differentiable nowhere. In short, FQPSO reaches the global optimum on 10 and 30 dimensions. In Fig 2 and Tables 6 and 7, it can be observed that except FQPSO with orders 0.1 and 0.2, FQPSO can always achieve better results than QPSO. Meanwhile, for function 6, 7 and 8, the convergence accuracies are better than QPSO when 0.3≤*α*≤0.9.

**Table 8:** Time consumption.

	*f*_1_	*f*_2_	*f*_3_	*f*_4_	*f*_5_	*f*_6_	*f*_7_	*f*_8_
**QPSO**	0.8123	0.9245	0.8155	0.8934	0.8688	0.9355	0.9642	0.9942
**FQPSO**	0.8471	0.9334	0.8358	0.9548	0.8899	0.9674	0.9856	1.032

In summary, FQPSO has superior ability in tackling multimodal functions compared with other algorithms. We can always find an appropriate fractional order for the algorithm that has better convergence accuracy than the integer order one in Group 2.

Table 8 shows the time consumption of FQPSO and QPSO in solving function optimization problems. The default time unit is seconds. The experimental results also confirm that the fractional order method only consumes a little more time in each iteration process, and does not cause a lot of waste of time.

### Compare with other variants of PSO

In this experiment, the best results of the FQPSO methods were used for comparison with other variants of PSO, including PSO, QPSO, CCPSO, NPSO and MRPSO. The parameters of the compared algorithms were set according to the recommendations in their original papers. The maximum numbers of FEs were respectively set to 10000 and 30000 for solving 10-D and 30-D problems. All experiments were performed with a population size of 20.

**Fig 3:**
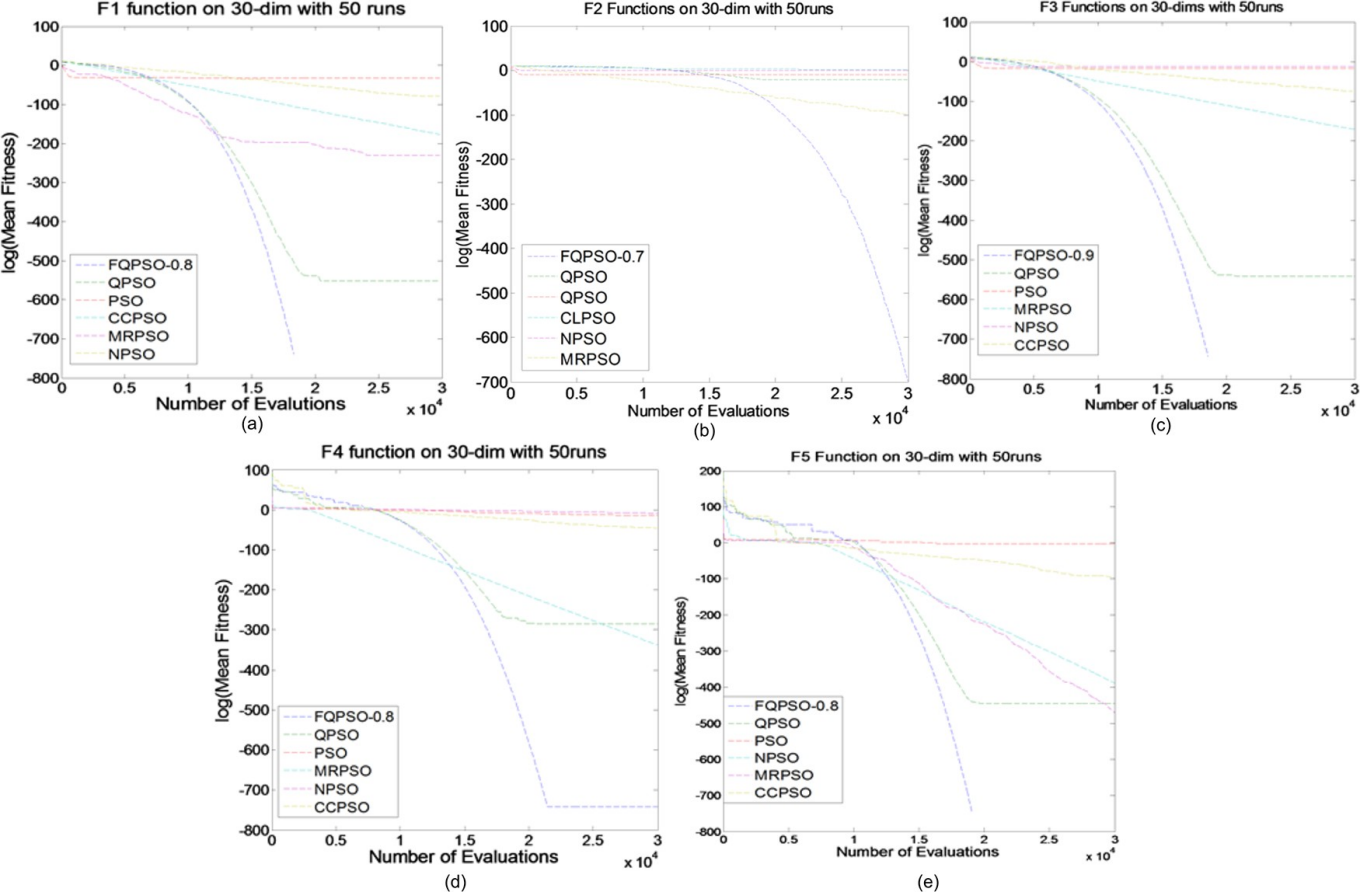
Comparison between different PSO algorithms on Group 1. (a) *f*_1_, (b) *f*_2_, (c) *f*_3_, (d) *f*_4_, (e) *f*_5_.

**Fig 4:**
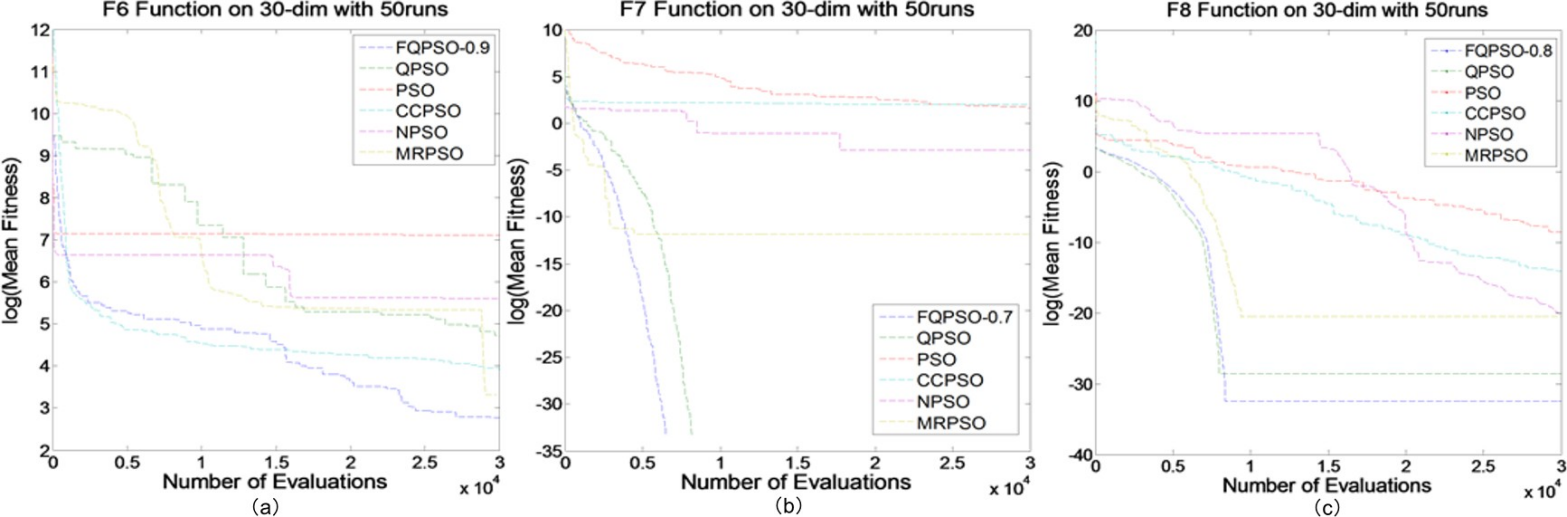
Comparison between different PSO algorithms on Group 2. (a) *f*_6_, (b) *f*_7_, (c) *f*_8_.

Tables 9 and 10 shows the statistical results of different algorithms on unimodal functions. From the previous results in the last subsection, we can see that FQPSO with *D*^0.8^ obtained the best results on functions 1–3 and FQPSO with *D*^0.7^ achieved the best results on functions 4–5. We fixed the orders to compare those results with other variants of PSO. The results from different algorithms on these five unimodal functions suggest that FQPSO is better at a fine-gained search than all the other algorithms. The rapid convergence of the FQPSO can be seen as an evidence for our observation in Fig 3. In summary, FQPSO performs best in solving unimodal functions among all the algorithms. Tables 9 and 10 and Fig 4 show the performances of different algorithms on Group 2.

**Table 9:** Comparison between different PSO algorithms on function 1–3.

Algorithm		*f*_1_		*f*_2_		*f*_3_	
		Dim = 10 FEs = 10000	Dim = 30 FEs = 30000	Dim = 10 FEs = 10000	Dim = 30 FEs = 30000	Dim = 10 FEs = 10000	Dim = 30 FEs = 30000
**FQPSO**	Best Mean std	0 0 0	0 0 0	0 0 0	1.6812e-313 1.9619e-296 5.3432e-307	0 0 0	0 0 0
**QPSO**	Best Mean std	5.6324e-267 4.5321e-265 3.4523e-266	2.3453e-241 1.5837e-239 6.3245e-241	3.7568e-258 1.8379e-255 2.5431e-256	1.7033e-237 6.8877e-234 2.9832e-237	1.5e-256 8.1e-254 5.31e-256	1.01e-243 1.86e-241 7.543e-243
**PSO**	Best Mean std	7.764e-20 1.17e-20 6.3e-20	6.7954e-14 1.58e-13 4.17e-13	5.8742e-16 4.9000e-15 1.3864e-16	4.3257e-10 1.2264e-09 2.4987e-10	5.8734e-21 9.3854e-20 3.5467e-19	3.876e-12 2.086e-06 6.4303e-11
**CCPSO**	Best Mean std	3.2341e-97 1,2313e-95 2.8734e-97	7.4324e-86 6.0851e-84 5.3241e-85	1.2353e-20 8.3483e-20 5.9834e-20	9.3557e-16 1.9619e-13 5.7934e-15	6.3258e-43 8.5423e-42 1.5425e-43	5.2134e-35 3.6880e-33 7.3424e-34
**NPSO**	Best Mean std	3.4653e-53 5.2356e-52 2.3456e-53	5.3789e-38 4.8357e-36 9.2134e-37	3.4453e-22 9.5151e-18 2.3456e-21	5.2223e-13 1.2580e-11 9.9863e-13	1.7431e-14 3.4564e-14 2.6731e-14	9.3452e-09 7.2875e-06 8.4245e-08
**MRPSO**	Best Mean std	1.5677e-110 1.239e-109 8.2345e-110	5.8723e-97 6.2391e-93 6.3394e-97	6.3434e-61 3.5639e-48 9.5332e-51	4.9053e-45 4.4788e-44 7.3421e-44	3.4546e-85 9.4671e-84 2.3546e-85	3.4546e-85 9.4671e-84 2.3546e-85

**Table 10:** Comparison between different PSO algorithms on function 4–6.

Algorithm		*f*_4_		*f*_5_		*f*_6_	
		Dim = 10 FEs = 10000	Dim = 30 FEs = 30000	Dim = 10 FEs = 10000	Dim = 30 FEs = 30000	Dim = 10 FEs = 10000	Dim = 30 FEs = 30000
**FQPSO**	Best Mean std	0 0 0	0 0 0	0 0 0	0 0 0	0 0 0	7.0106e-06 1.4384 1.234
**QPSO**	Best Mean std	1.4622e-248 1.2011e-215 5.6231e-235	6.2412e-146 1.2203e-137 4.562e-137	9.397e-189 3.5943e-185 6.3423e-187	1.1027e-154 1.1027e-155 6.3453e-155	1.7764e-10 2.1453e-08 5.4214e-09	15.9395 16.0250 5.3453
**PSO**	Best Mean std	5.324e-13 1.5242e-11 2.584e-11	1.0000e-09 7.5267e-07 6.3324e-08	5.4234e-19 2.534e-18 3.4532e-19	5.324e-07 0.0571 0.000424	4.34e-06 3.2e-05 3.2e-05	7.3474 7.8363 1.3456
**CCPSO**	Best Mean std	1.324e-30 8.3453e-27 4.5356e-29	7.4352e-23 6.3257e-21 5.6485e-22	6.4234e-55 4.5313e-51 8.5423e-54	5.3256e-44 9.4075e-42 3.4578e-43	0.1234 0.1423 0.0542	2.9768 4.475 6.4246
**NPSO**	Best Mean std	5.6354e-09 4.3563e-07 2.3446e-09	7.5242e-07 1.8913e-04 4.6452e-06	6.4312e-183 4.5683e-180 9.4563e-182	4.5353e-175 2.2115e-169 3.4532e-172	0.34561 0.40593 0.5289	5.323 5.354 0.0034
**MRPSO**	Best Mean std	6.4232e-154 7.4323e-151 4.2456e-154	5.3133e-150 2.9806e-147 4.5624e-149	8.6431e-212 6.4331e-210 6.7456e-210	4.3534e-209 1.0762e-203 3.5356e-206	2.456e-08 4.355e-06 5.342e-07	4.567 4.678 0.543

Tables 9 and 10 shows the statistical results of different algorithms on unimodal functions. From the previous results in the last subsection, we can see that FQPSO with *D*^0.8^ obtained the best results on functions 1–3 and FQPSO with *D*^0.7^ achieved the best results on functions 4–5. We fixed the orders to compare those results with other variants of PSO. The results from different algorithms on these five unimodal functions suggest that FQPSO is better at a fine-gained search than all the other algorithms.

The rapid convergence of the FQPSO can be seen as an evidence for our observation in Fig 3.

In summary, FQPSO performs best in solving unimodal functions among all the algorithms. Tables 10 and 11 and Fig 4 show the performances of different algorithms on Group 2.

**Table 11:** Comparison between different PSO algorithms on function 7–8.

Algorithm		*f*_1_		*f*_2_	
		Dim = 10 FEs = 10000	Dim = 30 FEs = 30000	Dim = 10 FEs = 10000	Dim = 30 FEs = 30000
**FQPSO**	Best Mean std	7.1054e-15 9.9476e-15 8.4534e-14	0 0 0	7.6438e-34 5.3222e-29 7.4345e-33	1.6409e-15 5.1514e-15 3.4356e-15
**QPSO**	Best Mean std	1.9257e-35 1.8609e-33 4.3456e-34	2.5251e-26 3.6749e-21 5.4245e-26	1.7059e-19 6.8877e-20 7.4352e-19	7.9936e-15 7.9936e-15 0
**PSO**	Best Mean std	4.9016e-08 8.345e-06 5.4345e-07	41.3 45.043 10.413	5.6534e-14 6.4523e-13 4.5634e-14	5.4326e-05 7.5331e-04 4.5674e-05
**CCPSO**	Best Mean std	8.3079e-05 1.1100e-04 4.5634e-05	0.0035 3.8253 6.4231	6.5341e-19 3.2145e-18 5.6313e-19	7.4243e-09 4.4509e-08 6.4234e-09
**NPSO**	Best Mean std	1.7431e-14 3.4564e-14 2.6731e-14	3.4531e-05 0.0801 0.0004	3.4356e-22 5.3561e-19 5.5546e-22	5.3563e-13 8.4356e-13 6.4325e-13
**MRPSO**	Best Mean std	6.5183e-11 9.7154e-09 4.5623e-10	1.6486e-08 4.678e-05 6.3245e-07	5.6356e-27 1.2343e-24 3.4465e-26	6.4382e-14 9.3435e-14 7.4231e-14

From the previous results in the last subsection, it can be noticed that FQPSO with *D*^0.9^ obtained the best result on function 6, FQPSO with *D*^0.7^ got the best result on function 7, and FQPSO with *D*^0.9^ achieved the best result on function 8. We also fixed the orders to compare those results with other variants of PSO. It can be seen that FQPSO obtains the global optimum on 10 and 30 dimensions. FQPSO is better to deal with multimodal functions than other algorithms.

In the results of different PSOs on 30 dimensions also supports our conclusion that FQPSO is suitable for multimodal functions. In summary, FQPSO performs best in solving both unimodal and multimodal functions among all the algorithms.

## Conclusion

Inspired by the properties of fractional calculus, we presented a novel QPSO algorithm incorporated with fractional calculus strategy, which is based on the properties of long time memory and non-locality of fractional calculus. The goal is to employ the proposed method to accelerate not only the convergence speed but also avoid the local optimums. Since the property of fractional calculus enables quantum-particles in FQPSO to appear anywhere during iterations, it significantly improves the global searching ability. Furthermore, FQPSO also increases the convergence rate for the quantum particles. As a result, the proposed FQPSO method achieves more favorable results than all the other algorithms.
